# Inflammatory Cytokines Alter Mesenchymal Stem Cell Mechanosensing and Adhesion on Stiffened Infarct Heart Tissue After Myocardial Infarction

**DOI:** 10.3389/fcell.2020.583700

**Published:** 2020-10-23

**Authors:** Dan Zhu, Peng Wu, Changchen Xiao, Wei Hu, Tongtong Zhang, Xinyang Hu, Wei Chen, Jian’an Wang

**Affiliations:** ^1^Department of Cardiology of the Second Affiliated Hospital, Zhejiang University School of Medicine, Hangzhou, China; ^2^Department of Pharmacology, Zhejiang University School of Medicine, Hangzhou, China; ^3^Key Laboratory for Biomedical Engineering of the Ministry of Education, College of Biomedical Engineering and Instrument Science, Zhejiang University, Hangzhou, China

**Keywords:** cytokine, mechanosensing, adhesion, mesenchymal stem cell, myocardial infarction

## Abstract

Mesenchymal stem cell (MSC) transplantation has demonstrated its potential in repairing infarct heart tissue and recovering heart function after myocardial infarction (MI). However, its therapeutic effect is still limited due to poor MSC engraftment at the injury site whose tissue stiffness and local inflammation both dynamically and rapidly change after MI. Whether and how inflammatory cytokines could couple with stiffness change to affect MSC engraftment in the infarct zone still remain unclear. In this study, we characterized dynamic stiffness changes of and inflammatory cytokine expression in the infarct region of rat heart within a month after MI. We found that the tissue stiffness of the heart tissue gradually increased and peaked 21 days after MI along with the rapid upregulation of tumor necrosis factor-α (TNF-α), interleukin-6 (IL-6), and interleukin-1β (IL-1β) in the first 3 days, followed by a sharp decline. We further demonstrated *in vitro* that immobilized inflammatory cytokine IL-6 performed better than the soluble form in enhancing MSC adhesion to stiffened substrate through IL-6/src homology 2 (SH2) domain-containing tyrosine phosphatase-2 (SHP2)/integrin signaling axis. We also confirmed such mechano-immune coupling of tissue stiffness and inflammatory cytokines in modulating MSC engraftment in the rat heart after MI *in vivo*. Our study provides new mechanistic insights of mechanical–inflammation coupling to improve MSC mechanosensing and adhesion, potentially benefiting MSC engraftment and its clinical therapy for MI.

## Introduction

Acute myocardial infarction (AMI) is a leading cause of human mortality worldwide. It triggers an acute inflammatory response and tissue fibrosis that significantly stiffen the infarct tissue of the heart, impairing the contraction ability of the myocardium and heart function (Liehn et al., 2011; Frangogiannis, 2014). Most clinically approved treatments for MI only modulate hemodynamics to reduce early mortality but are unable to repair infarcted myocardium or recover heart function to reduce the following incidence of heart failure (Ranganath et al., 2012; Miao et al., 2017).

In recent decades, mesenchymal stem cell (MSC) transplantation has demonstrated its potential for repairing infarcted myocardium and restoring heart function through its anti-inflammation and anti-fibrosis functions (Miao et al., 2017). However, its therapeutic efficacy observed in randomized controlled clinical trials has been limited. A meta-analysis reported that an increase of the left ventricular ejection fractions (LVEFs) with MSC therapy by only 2.92% and reducing the incidence of heart failure in response to the therapy is not significantly better compared to revascularization procedure [percutaneous coronary intervention (PCI) or thrombolysis or coronary artery bypass surgery] (Afzal et al., 2015). It was reported that only a small percentage of MSCs are retained at the injury site after transplantation (Lee et al., 2009). This low MSC engraftment rate could be one of the major obstacles in enhancing the efficacy of MSC-based therapy (Sanganalmath and Bolli, 2013). In fact, several approaches have been developed to enhance MSC engraftment. For example, preconditioning MSCs with hypoxia has been shown to maintain cell viability and improve MSC engraftment through the leptin signaling pathway (Hu et al., 2014). Although pro-survival strategies have been proven to be effective, they still have not solved the problem of poor adhesion of MSCs to the extracellular matrix (ECM), which is the prerequisite for a successful engraftment of MSC therapy.

Cell adhesion to the ECM is modulated by mechanical properties of ECM, which change dynamically after the onset of AMI due to the pathological process of inflammation and tissue fibrosis (Discher et al., 2005; Trichet et al., 2012; Elosegui-Artola et al., 2016). Cells probe substrate stiffness through mechanosensing machinery that involves integrins, focal adhesion (FA) proteins, and the actomyosin cytoskeleton (Roca-Cusachs et al., 2012; Elosegui-Artola et al., 2016), among which integrin’s binding with ECM provides the main mechanical linkage. In response to matrix stiffness increase, cells generate larger force, which not only strengthens the integrin/ECM binding through “catch bond” but also induces clustering of integrin to promote adhesion development (Kong et al., 2009; Roca-Cusachs et al., 2009; Changede et al., 2015). Stiffness of the infarct region along with the progression of heart remodeling after MI was reported to range from several kPa to tens of MPa (Berry, 2006; Zhang et al., 2011; Arunachalam et al., 2018; Rusu et al., 2019), which may differentially promote MSC adhesion and engraftment.

Other than remodeling of mechanical properties of the infarct region, inflammatory cytokines also dynamically change after MI. The three main inflammatory cytokines, including tumor necrosis factor-α (TNF-α), interleukin-1β (IL-1β), and interleukin-6 (IL-6), have been reported to be upregulated after MI (Frangogiannis, 2014; Hu et al., 2016). In addition to its soluble form, TNF-α can also bind to the N-terminal of fibronectin (FN) to be an immobilized form, which augments integrin β_1_-mediated adhesion of CD4^+^ T lymphocytes to FN (Alon et al., 1994). *In vivo*, pretreating MSCs with TNF-α enhances MSC engraftment into the infarcted heart (Kim et al., 2009). Phosphatase SHP2, which is activated upon IL-6 stimulation (Sun et al., 2017), has been reported to promote FA growth in response to matrix stiffness (Lee et al., 2013). However, whether and how these inflammatory cytokines integrate with tissue stiffness to cooperatively regulate MSC adhesion to the infarct heart still remain unclear.

In the present study, we integrated *in vitro* biomechanical assays along with *in vivo* characterization and revealed that MSC adhesion could be enhanced by stiffened substrate and that immobilized inflammatory cytokine IL-6 can further strengthen MSC adhesion through IL-6/SHP-2/integrin signaling axis. We also demonstrated that enhancement of MSC engraftment *in vivo* after MI temporally coincides with cytokine upregulation and tissue stiffness increase, respectively, confirming the role of mechanical–chemical coupling in the modulation of MSC engraftment.

## Materials and Methods

### Animals

All animal experiments were conducted in accordance with the Guide for the Care and Use of Laboratory Animals published by the United States National Institutes of Health (National Institutes of Health publication no. 85-23, revised 1996) and were approved by the Institutional Animal Care and Use Committee of Zhejiang University. Male Sprague-Dawley rats were purchased from Shanghai Slac Laboratory Animal Technology Corporation. The animals were fed a standard laboratory diet and maintained under a 12-h light/dark cycle.

### Acute Myocardial Infarction Induction

Male Sprague-Dawley rats (weighing 250–300 g) were anesthetized with chloral hydrate (35 mg/kg) by intraperitoneal injection, mechanically ventilated, and left thoracotomy performed to expose the left anterior descending (LAD) coronary artery. MI was induced by permanent ligation of the LAD coronary artery using a 6-0 silk suture, and regional myocardial ischemia was observed by a rapid discoloration over the at-risk area. After induction of MI, the rats’ thoraces were closed and the rats recovered.

### Atomic Force Microscopy Characterization

**FIGURE 1:**
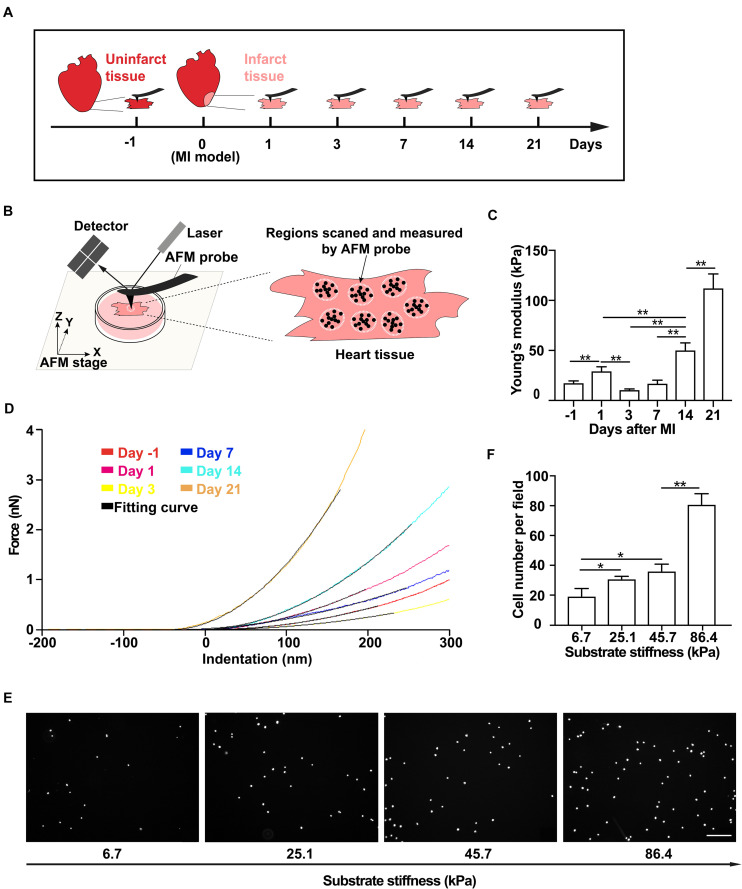
Tissue stiffness of the infarct region of the rat heart increases after myocardial infarction (MI) and promotes mesenchymal stem cell (MSC) adhesion. **(A–D)** Schematics of the measurement time course of rat heart tissue stiffness by atomic force microscopy (AFM) **(A)** and the illustration of AFM micro-indentation to measure rat heart tissue stiffness by AFM **(B)** after MI, of which are the representative force-indentation curves **(D)** and means of tissue stiffness **(C)**. Tissue from the infarct region was cut into small blocks for AFM probing. At least 10 varied positions over the tissue block surface were measured, and 10–15 indentations were made on each position. White dotted circles and black dots in panel **(B)** indicate randomly selected positions and different indentations, respectively. Dashed lines in panel **(D)** are the best fit to corresponding stiffness measurements by AFM with the Hertz Cone model. **(E,F)** Representative images **(E)** and average numbers **(F)** of the attached MSCs after wash assay on PAA gel substrates with different stiffnesses that mimic infarct regions after MI. Scale bar in panel **(E)** refers to 200 μm. ** and * in panels **(C,F)** refer to *p* < 0.01 and *p* < 0.05, respectively. Error bars in panels **(C,F)** represent SEM of three repeats.

Hearts of rats were obtained at 1, 3, 7, 14, and 21 days after MI to perform atomic force microscopy (AFM) measurements of elasticity in the infarct region. Tissue from the infarct region was cut into small blocks, each of which was about 10 mm (length) × 5 mm (width) × 1 mm (thickness) and was then attached to poly-L-lysine-coated coverslips in Dulbecco’s modified Eagle’s medium (DMEM) and mounted on the AFM (Asylum Research, Santa Barbara, CA, United States) sample stage for probing. The tissue blocks were indented with a blunted pyramid-tipped cantilever (TR400PSA, Olympus, Tokyo, Japan) at rates <2 μm/s to avoid detection of viscoelastic properties of cells and the ECM. The spring constant (60–80 pN/nm) and deflection sensitivity of the cantilever were calibrated before each round of micro-indentation experiments. To obtain data over the entire sample surface, at least 10 different positions of the tissue blocks were measured, and 10–15 indentations were made on each position (Figure 1B). Each force-indentation curve was fitted to a Hertz cone model to determine an elastic modulus, and the elastic moduli were averaged for each rat.

### Mesenchymal Stem Cell Culture

Mouse MSCs were obtained from Cyagen Biosciences (Guangzhou, China) and cultured in DMEM (Corning, Manassas, VA, United States) supplemented with 10% fetal bovine serum (FBS) (Invitrogen, Carlsbad, CA, United States) at 37°C in a 5% CO_2_ incubator. The characteristic surface marker expressions of MSCs were confirmed with flow cytometry before the experiments (Supplementary Figures 1B–E).

### Cell Preparation and Transplantation

Before transplantation, MSCs were engineered to express ZsGreen *via* lentiviral transfection as described previously (Zhu et al., 2018). Briefly, lentivirus particles were purchased from GeneChem, Shanghai, and the transduction efficiency was evaluated by fluorescence microscopy. One, 3, 7, 14, and 21 days after MI, the thoraces of the rats (six animals in each group) were reopened to perform intramyocardial cell transplantation. Each rat was injected with 1 × 10^7^ MSCs in 1 ml DMEM from five sites (2 × 10^6^ cells/site) in the infarct region *via* a Hamilton syringe. Twenty-four hours later, hearts were collected, and cell engraftment was analyzed with quantitative polymerase chain reaction (qPCR) and fluorescence microscopy.

### Quantitative Analysis of Cell Engraftment

The number of engrafted cells in the heart was determined *via* qPCR assessments of ZsGreen DNA levels as described previously (Hu et al., 2014). Genomic DNA was prepared from cells and tissues according to the manufacturer’s instructions of TAKARA MiNiBEST Universal Genomic DNA Extraction Kit (#9765, Takara Biotechnology Co., Dalian, China). The purified DNA was amplified with ZsGreen forward (5′ GCGAGAAGATCATCCCCGTG 3′) and reverse (5′ ACTTCTGGTTCTTGGCGTCG3′) primers by using SYBR Premix Ex Taq (#RR420, Takara Biotechnology Co., Dalian, China). Absolute standard curves were generated from serial dilution of the genomic DNA of a known number of MSCs (diluting the genomic DNA of ZsGreen-positive MSCs in normal heart tissue genomic DNA at a ratio from 1:1 to 1:1,000) and served as the reference for calculating the number of engrafted cells. The engraftment rate was determined from the ratio between engrafted cell number and injected cell number.

### Immunohistochemical Analysis

The whole hearts were dehydrated in 30% sucrose solution, embedded in Tissue-Tek OCT compound (Sakura Finetek USA, Inc., Torrance, CA, United States), and snap frozen in liquid nitrogen. Heart tissue from the infarct region (site of cell injection) was sliced to obtain frozen sections of 6.0 μm thick. The slides were fixed in 4% paraformaldehyde, permeabilized in 0.2% Triton X-100, blocked with 5% bovine serum albumin (BSA), and incubated with different primary antibodies, including anti-α-actinin antibody (#ab9465, Abcam, Cambridge, MA, United States), anti-CD68 antibody (#ab31630, Abcam, Cambridge, MA, United States), anti-IL-1β antibody (#ab9722, Abcam, Cambridge, MA, United States), anti-IL-6 antibody (#LS-C746886, Biocompare, San Francisco, CA, United States), and anti-TNFα antibody (#ab6671, Abcam, Cambridge, MA, United States) overnight at 4°C. Then, the slides were incubated with the cognate secondary antibodies for 1 h at room temperature (RT). Sections incubated with only secondary antibody were used as negative control for non-specific staining. Nuclei were stained with Hoechst 33258 (Invitrogen, Carlsbad, CA, United States). After the staining, the sections were mounted on a fluorescent microscope for examination. Images were taken at a magnification of 600×, and the number of CD68^+^ or CD68^+^cytokine^+^ cells from each high-power field (HPF) image was calculated and averaged for each rat.

### Preparation of Polyacrylamide Gels

Glass-bottom dishes were activated with 2% 3-aminopropyltrimethoxysilane (Sigma-Aldrich, St Louis, MO, United States) in acetone, washed three times with ddH_2_O, and air-dried for 10 min. Stock solutions were prepared from a mixture of 40% acrylamide and 2% bis-acrylamide and optimized for preparation of polyacrylamide (PAA) gels of different stiffness (see Supplementary Table). Working solutions containing the final desired concentrations of acrylamide/bis-acrylamide were obtained from stock solutions (see Supplementary Table). Ammonium persulfate (APS) and tetramethylethylenediamine (TEMED) were added to initiate gel polymerization. Then, 8–10 μl of the acrylamide solution were added to the center of glass-bottom dishes and covered with 12-mm-diameter glass coverslips. After gel polymerization, top coverslips were removed. Then, 200 μl of Sulfo-SANPAH (Thermo Scientific, Waltham, MA, United States) were applied to the gel surface and exposed to UV light. After UV activation, Sulfo-SANPAH changed its color from orange to brown. The gel surface was then washed and incubated with 20 μg/ml of FN (Sigma-Aldrich, St. Louis, MO, United States) overnight at 4°C. PAA gels were washed three times with sterile phosphate-buffered saline (PBS) and sterilized under a germicidal lamp for 2 h.

### Wash Assay

Mesenchymal stem cells were trypsinized and resuspended at 2 × 10^5^ cells/ml in DMEM with 10% FBS. Then, 1 ml of cell suspension was added to each dish from different groups. The dishes were incubated at 37°C for 30 min to allow the cells to adhere to the surface and washed three times with PBS to remove any non-adherent cells. After washing, DMEM with 10% FBS were added to each dish, and the cells were incubated at 37°C for 4 h for recovery. After cell recovery, the cells were fixed in 4% paraformaldehyde, permeabilized in 0.2% Triton X-100, and stained with Hoechst 33258 (Invitrogen, Carlsbad, CA, United States). Fluorescent images were acquired at a magnification of 400×, and cell number from each image was counted and averaged for each dish of the different groups.

### Cell Adhesion Under Flow

Fibronectin and octadecyl rhodamine B chloride (R18) were obtained from Sigma-Aldrich (St. Louis, MO, United States) and Invitrogen (Carlsbad, CA, United States), respectively. IL-1β, IL-6, and TNF-α were obtained from Sino Biological (Beijing, China). Adhesive substrates were prepared from perfusion of microfluidic channels in the BioFlux plate with different concentration of FN at 5 dyn/cm^2^ for 15 min, followed by incubation at RT for 1 h, and blocked with 0.5% BSA at 5 dyn/cm^2^ for 15 min. All cytokine-containing adhesive substrates were prepared from perfusion of cytokines together with FN. MSCs were trypsinized, stained with 300 nM R18, resuspended at 1 × 10^6^ cells/ml in PBS, and perfused from the inlet wells for 10 min at 10 dyn/cm^2^. In experiments that assess the effect of soluble cytokines, cells were incubated with different cytokines for 30 min at RT before perfusion. Images of attached cells after the perfusion were captured at a magnification of 100× using a Nikon TS100 microscope (Nikon Instruments, Inc., Melville, NY, United States) equipped with a CCD camera (QICAM, QImaging, Surrey, British Columbia) and the BioFlux 200 software. Cell number from each image was counted and averaged for each group.

### Flow Cytometry and Western Blotting

For cell surface marker characterization, MSCs were stained with anti-CD45 FITC (#553080, BD Biosciences, San Jose, CA, United States), anti-Stem-cell antigen 1 FITC (SCA-1; #11-5981-85, eBioscience, San Diego, CA, United States), anti-CD44 PE (#12-0441-81, eBioscience, San Diego, CA, United States), and anti-CD31 PE (#553373, BD Biosciences, San Jose, CA, United States) for 15 min at RT and analyzed by flow cytometry after washing with PBS. For integrin β1 and β3 expression determination, MSCs (1 × 10^5^ cells/group) untreated or stimulated with FN, IL-6, and FN + IL-6 were digested with trypsin and stained with anti-integrin β1 PE (#102215, BioLegend, San Diego, CA, United States) or β3 antibody (#104305, BioLegend, San Diego, CA, United States) and further analyzed with flow cytometry. For Western blotting, MSCs (1 × 10^6^ cells/group) untreated or stimulated with FN, IL-6, and FN + IL-6 were digested and lysed with 80 μl lysis buffer containing 2% NP-40 and 1× protease inhibitor cocktail (#B14001, BioTool, Switzerland). The samples were heated for 5 min at 95°C after adding 20 μl 5× loading buffer. Equal volumes of samples were separated *via* electrophoresis on 10% sodium dodecyl sulfate (SDS)-polyacrylamide gel electrophoresis (PAGE) gels, transferred onto polyvinylidene fluoride (PVDF) membranes (Millipore, Billerica, MA, United States), and blocked with 5% (w/v) non-fat dry milk (BD Biosciences, Franklin Lakes, NJ, United States) in Tris-buffered saline with 0.1% Tween20 for 1 h at RT. The membranes were incubated with primary antibodies against β-actin (#KC-5A08, Aksomics, Chengdu, China) and SHP2 (#sc-7384, Santa Cruz, CA, United States) overnight at 4°C. The blots were then incubated with the appropriate horseradish peroxidase-conjugated secondary antibodies and visualized with an enhanced chemiluminescence (ECL) system (Millipore, Boston, MA, United States).

### Statistical Analysis

Data are presented as mean ± SEM. Two-tailed Student’s *t*-test was used when two groups were compared, and analysis of variance (ANOVA) tests were applied when more groups were analyzed. All statistical analyses were performed with the SPSS software, version 17.0. Differences were considered statistically significant at *p* < 0.05.

## Results

### Tissue Stiffness of the Infarct Region of the Rat Heart Increases Gradually Within 21 Days After Myocardial Infarction

To determine the tissue stiffness of the infarct region of the rat heart, we collected the heart tissues on day 1 from normal groups and on days 1, 3, 7, 14, and 21 from MI groups (Figure 1A and Supplementary Figure 1A). We then used AFM indentation assay to quantitatively measure the stiffness of non-infarcted rat heart tissue and the infarct region of the rat heart on different days after MI (Figure 1B). We found that the stiffness of non-infarcted tissue is 17.4 ± 2.2 kPa before MI (Figures 1C,D), similar to previous reported values (Berry, 2006). After MI, the stiffness of the infarcted tissue increased to 29.2 ± 4.5 kPa on day 1 and slightly decreased to 10.5 ± 1.1 kPa on day 3, followed by rapid and progressive increase to 112.0 ± 14.5 kPa from day 7 to day 21 (Figures 1C,D). These data demonstrate that the stiffness of the infarct region of rat heart after MI dynamically changes, that is, gradually becoming stiffer along with MI progression in 21 days.

### Increased Substrate Stiffness Promotes Mesenchymal Stem Cell Adhesion *in vitro*

As MSC adhesion is the critical step toward a successful transplantation, we next tested whether the stiffened tissue substrate would affect MSC adhesion. Using mouse MSC cell line (Supplementary Figures 1B–E), we performed wash assay to wash away pre-adhered MSCs on FN-coated substrates. These substrates were made of PAA gels to mimic various stiffnesses of the infarct region of the rat heart after MI. We found that the number of pre-adhered cells remained attached after washing increased with substrate stiffness increase (Figures 1E,F), demonstrating that the stiff substrate promotes MSC adhesion *in vitro*.

### Rapid Upregulation of Inflammatory Cytokines in the Infarct Region of the Rat Heart in the First 3 Days After Myocardial Infarction

**FIGURE 2:**
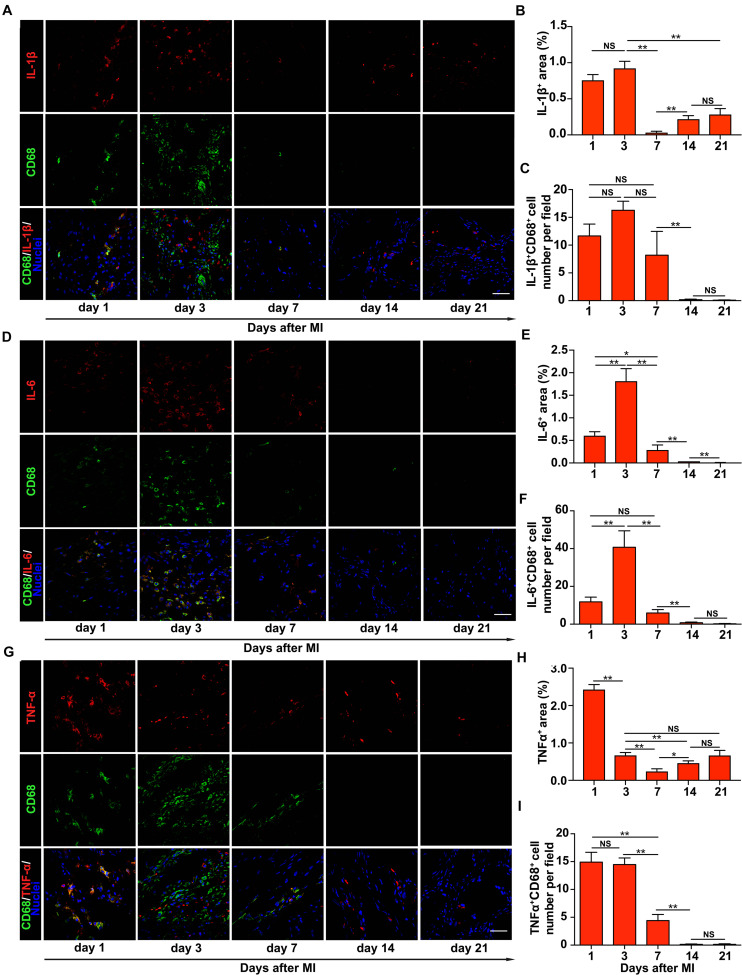
Upregulation of inflammatory cytokines in the infarct region of the rat heart early after myocardial infarction (MI). Representative images of immunohistochemical staining of interleukin-1β (IL-1β) **(A)**, interleukin-6 (IL-6) **(D)**, and tumor necrosis factor-α (TNF-α) **(G)** in the infarct region of the rat heart at different times after MI. Comparisons of the numbers of IL-1β^+^CD68^+^ **(B)**, IL-6^+^CD68^+^ **(E)**, and TNF-α^+^CD68^+^ **(H)** cells per field, respectively, and the percentage of the surface area within the infarct region that was stained positively for IL-1β **(C)**, IL-6 **(F)**, and TNF-α **(I)**, respectively. In panels **(A,D,G)**, scale bars refer to 40 μm. **, *, and NS refer to *p* < 0.01, *p* < 0.05, and no significance, respectively. *N* = 3 rats for each time point. Error bars in panels **(B,C,E,F,H,I)** represent SEM of three repeats.

We next characterized the infiltration of macrophages and the expression of inflammatory cytokines in the infarct region on different days after MI. We performed the co-staining of α-actinin with IL-1β, IL-6, or TNF-α and found that inflammatory cytokines were not co-localized with cardiac tissues (Supplementary Figure 2). The result showed a rapid upregulation of IL-1β, IL-6, and TNF-α and infiltration of macrophage in the first 3 days after MI in the peri-infarct/infarct zone, followed by a sharp decline of the expression level of these three cytokines and the number of infiltrated macrophages (Figures 2A,D,G). These cytokines and macrophages declined to a low level on day 21 after MI (Figures 2B,E,H and Supplementary Figures 2, 3). In addition, we observed a very similar alteration of the number of macrophages producing these three inflammatory cytokines on different days after MI (Figures 2C,F,I), demonstrating that different from a progressive rise of the tissue stiffness of the infarct region, inflammatory cytokines and infiltrated macrophages are mainly upregulated early after MI.

**FIGURE 3:**
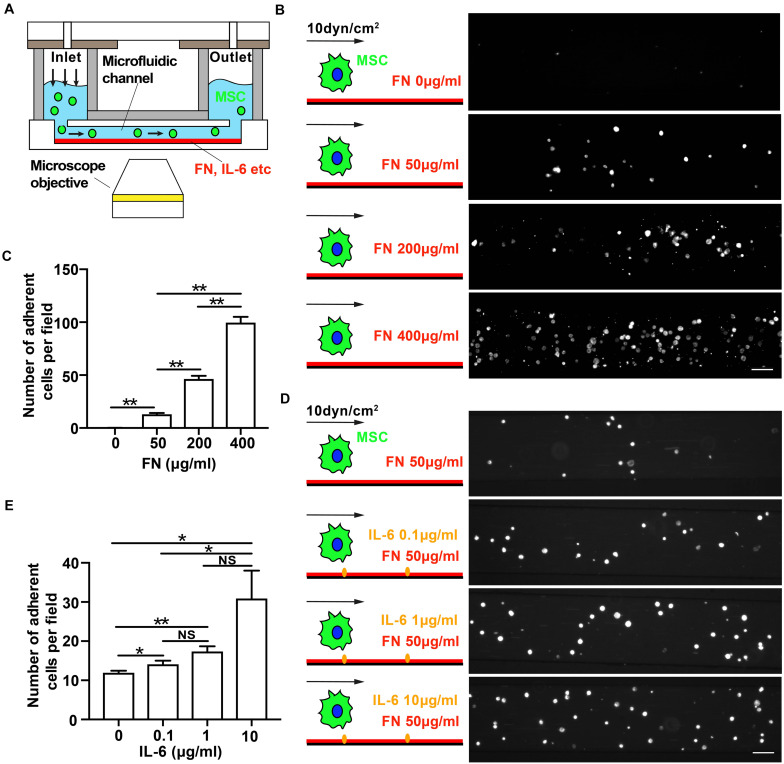
Immobilized interleukin-6 (IL-6) promotes mesenchymal stem cell (MSC) adhesion under shear flow. **(A)** Schematics of flow chamber assay to qualitatively measure shear force-dependent adhesion strength of MSCs to microfluidic channel’s substrate coated with fibronectin (FN) in the absence or presence of immobilized IL-6. **(B–E)** Representative images of attached MSCs after perfusion over microfluidic channels coated with increasing concentrations of FN **(B)** or IL-6 **(D)** at shear stress of 10 dyn/cm^2^. Quantitative results of average numbers of adherent MSCs per field are shown in panels **(C,E)**, respectively. Scale bars in panels **(B,D)** refer to 200 μm. **, *, and NS in panels **(D,E)** refer to *p* < 0.01, *p* < 0.05, and no significance, respectively. Error bars in panels **(D,E)** represent SEM of three repeats.

### Immobilized IL-6 Promotes Mesenchymal Stem Cell Adhesion Under Shear Flow

Whether the elevation of inflammatory cytokines early after MI affects MSC adhesion is unclear. To answer this question, we used flow chamber assay to determine the adhesion strength of MSC binding to FN-coated microfluidic channels under shear stress to examine the mechano-regulated MSC adhesion (Figure 3A). We at first verified and confirmed that the MSC adhesion was mainly mediated through specific binding of cells to substrate-coated FN as the number of attached MSCs increased with an increasing concentration of FN (Figures 3B,C). We then determined the effect of IL-1β, IL-6, and TNF-α on MSC adhesion under shear flow. We found that the pretreatment of MSC with soluble IL-6 or TNF-α had no effect on MSC adhesion to FN under shear flow, but pretreatment with soluble IL-1β inhibited MSC adhesion to FN under shear stress (Supplementary Figure 4).

Previous study reported that endothelium-presented but not soluble chemokines trigger instantaneous lymphocyte adhesiveness to endothelial cells (Shamri et al., 2005). So, we further determined whether immobilized (substrate-coated) cytokines would enhance MSC adhesion. Indeed, we found that the number of MSCs attached to FN increased with increasing concentration of immobilized IL-6 (Figures 3D,E). In contrast, immobilized IL-1β or TNF-α failed to do so (Supplementary Figure 5). Collectively, these results demonstrate that immobilized IL-6 promotes MSC adhesion under shear force, suggesting that mechanical force coupling with cytokine IL-6 signaling activates integrin to enhance MSC adhesion.

### The IL-6-Dependent Adhesion Enhancement on Mesenchymal Stem Cells Under Shear Force Is Mediated Through SHP2–Integrinα_5_β_1_ Signaling Axis

**FIGURE 4:**
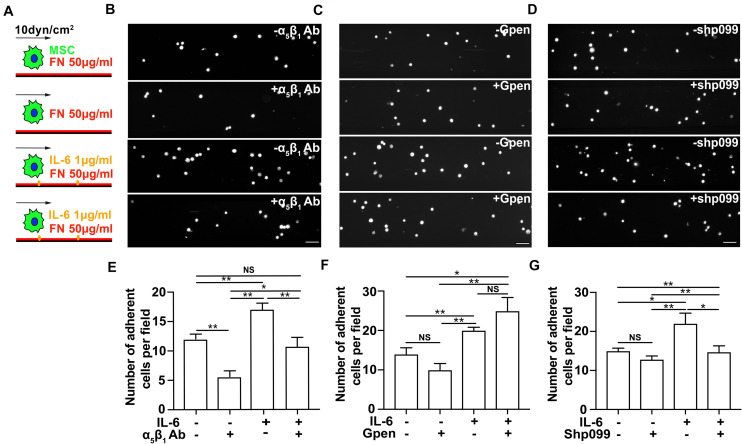
The interleukin-6 (IL-6)-dependent adhesion enhancement on mesenchymal stem cell (MSC) adhesion under shear is mediated through SHP2–integrinα5β1 signaling axis. Schematics **(A)** of MSC perfusion over microfluidic channels coated with 50 μg/ml of fibronectin (FN) in the absence (first and second rows in **B–D**) or in the presence of 1 μg/ml IL-6 (third and fourth rows in **B–D**), of which are their representative images of attached MSCs pretreated with α_5_β_1_ blocking mAb **(B)**, Gpen **(C)**, or shp099 **(D)** for 30 min before perfusion and are corresponding average numbers of adherent MSCs per field **(E–G)**, respectively. Scale bars in panels **(B–D)** refer to 200 μm. **, *, and NS in panels **(E–G)** refer to *p* < 0.01, *p* < 0.05, and no significance, respectively. Error bars in panels **(E–G)** represent SEM of three repeats.

It has been demonstrated that integrins α_5_β_1_ and α_v_β_3_, the FN-binding receptors, play different roles in the process of substrate mechanosensing (Roca-Cusachs et al., 2009; Schiller et al., 2013) and during MSC adhesion and migration (Docheva et al., 2007; Veevers-Lowe et al., 2011; Nitzsche et al., 2017). Integrin α_5_β_1_ probes the extracellular mechanical environment at the leading edge to search for appropriate adhesion sites (Galbraith et al., 2007) and determines the adhesion strength of cells to the ECM (Roca-Cusachs et al., 2009), while integrin α_v_β_3_ enables mechanotransduction that results in the directional orientation of the actin cytoskeleton and remodeling of FAs (Roca-Cusachs et al., 2009; Schiller et al., 2013). To further reveal the underlying mechanism of immobilized IL-6 in promoting MSC adhesion under shear force, we next tested whether IL-6 signaling activates integrins α_5_β_1_ and α_v_β_3_ binding to FN under force. As expected, blocking integrin α_5_β_1_ with its inhibitory antibody reduces the number of MSCs attached to FN-coated substrates under shear force in both the absence and presence of immobilized IL-6 (Figures 4A,B,E). In contrast, when cells were incubated with 0.5 mM of the cyclic GPenRGDSPCA (GPen) peptide to block α_v_β_3_, we did not observe any inhibitory effect on MSC adhesion in either the presence or the absence of immobilized IL-6 (Figures 4A,C,F). Collectively, these data demonstrate that immobilized IL-6 enhances integrin α_5_β_1_/FN binding-mediated MSC adhesion under shear force.

We next examined the underlying mechanism by which IL-6 signaling enhances integrin α_5_β_1_-mediated MSC adhesion from inside out. SHP2 has been reported as a key molecule in IL-6 signaling (Sun et al., 2017), and its deficiency impairs the maturation of cell–ECM adhesion (Lee et al., 2013; Sun et al., 2017). To determine whether SHP2 cross-linked IL-6 signaling to integrin α_5_β_1_, we thus inhibited SHP2 activity with an SHP2 allosteric inhibitor shp099 (Chen et al., 2016) and examined whether MSC adhesion to FN-coated substrates was affected by the presence or absence of immobilized IL-6. Interestingly, shp099 only reduced the number of MSCs adhering to FN-coated substrates in the presence of immobilized IL-6, but not in the absence of immobilized IL-6 (Figures 4F,G), suggesting that immobilized IL-6 signaling activates integrin α_5_β_1_ through SHP2 more efficiently than soluble IL-6 to enhance MSC adhesion. In conclusion, our results demonstrate that immobilized IL-6 promotes MSC adhesion through SHP2-integrinα_5_β_1_ signaling axis.

To further test whether the effect of immobilized IL-6 is mediated through upregulation of SHP2 and integrin, we performed flow cytometry and Western blot analysis of the expression levels of integrins β_1_ and β_3_ and SHP2, respectively, in the presence or absence of immobilized IL-6. Our results showed that immobilized IL-6 alone did not upregulate the expression of these molecules (Supplementary Figure 6). Collectively, along with the results that shp099 treatment abolished the enhancement effect of immobilized IL-6 (Figures 4A,D,G) on MSC adhesion, we speculate that conformational activation of SHP2 may serve as the downstream regulator of immobilized IL-6 to promote β_1_ integrin-dependent MSC adhesion.

### The Enhancement of Mesenchymal Stem Cell Engraftment *in vivo* After Myocardial Infarction Temporally Coincides With Cytokine Upregulation and Tissue Stiffness Increase

**FIGURE 5:**
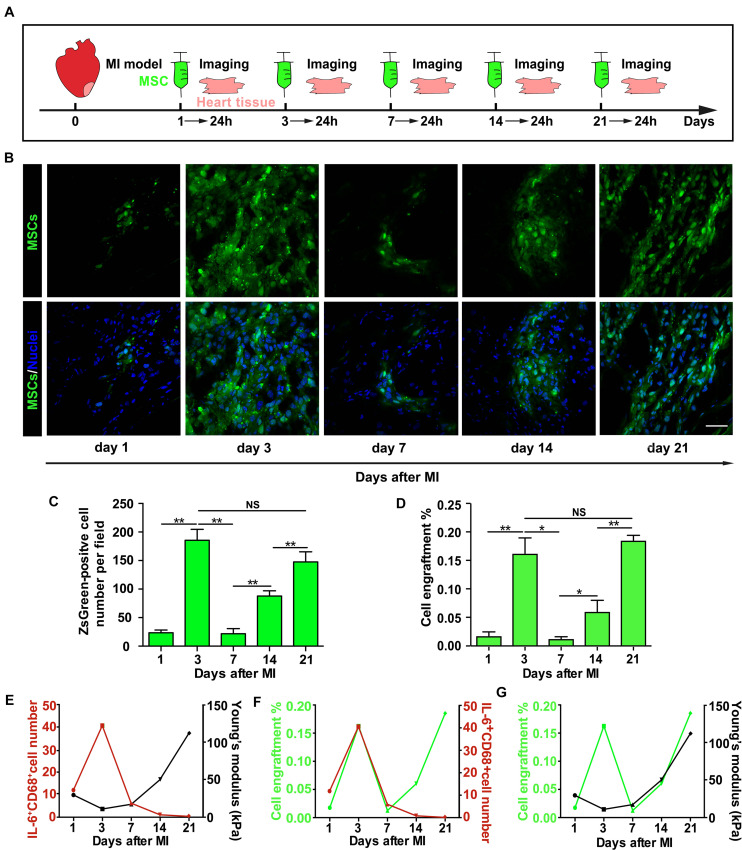
The enhancement of mesenchymal stem cell (MSC) engraftment *in vivo* after myocardial infarction (MI) temporally coincides with cytokine upregulation and tissue stiffness increase. **(A–C)** The illustration of experimental procedure of MSC transplantation, tissue harvest, and imaging of MSC engraftment **(A)**, of which are the representative fluorescence images of engrafted ZsGreen-labeled MSCs in the infarct region **(B)** and average numbers of engrafted MSCs per field **(C)**. qPCR measurements of ZsGreen gene level were used to confirm fluorescence microscopy examination, and the percentage of MSC engraftment **(D)** is calculated by dividing the number of adherent MSCs with total cell number administered in tissue samples from heart apex. **(E–G)** Correlation analyses of the number of IL-6^+^CD68^+^ cells with tissue stiffness of infarct region after MI **(E)** or of the number of IL-6^+^CD68^+^ cells with MSC engraftment **(F)** or of MSC engraftment with tissue stiffness of infarct region after MI **(G)**. Scale bars in panel **(B)** refer to 40 μm. **, *, and NS in panels **(C,D)** refer to *p* < 0.01, *p* < 0.05, and no significance, respectively. *N* = 3 rats for each time point. Error bars in panels **(C,D)** represent SEM of three repeats.

The dynamic changes of infarct tissue stiffness and inflammatory cytokine level after MI suggest that finding an optimal timing for MSC transplantation is essential for successful MSC engraftment. To determine how the injection timing of MSCs affects MSC function *in vivo*, especially MSC engraftment to the infarct region of the rat heart, we injected MSCs expressing ZsGreen into the infarct region of rat hearts on different days after MI (Figure 5A), collected the hearts 24 h after each injection, and characterized their engraftment efficiency. We found that only a few MSCs were retained in the infarct heart 1 day after MI (Figures 5B,C). However, the number of engrafted MSCs increased dramatically 3 days after MI, and 7 days after MI, it rapidly dropped to the level approximate to that on day 1. Since then, the number of engrafted MSCs gradually increased from day 7 to day 21 (Figures 5B,C). To confirm these results obtained from fluorescence microscopy examination, we further performed qPCR and found a very similar efficiency of MSC engraftment at different times after MI (Figure 5D). A detailed analysis of the results further reveals the temporal coincidence of highest MSC engraftment efficiency with highest IL-6 expression and tissue stiffness on day 3 and day 21, respectively (Figures 5E–G), indicating that both heart stiffness and inflammatory cytokines and their dynamic coupling are essential regulators for MSC adhesion and engraftment on the infarct region of rat hearts. Collectively, these results demonstrate that both day 3 and day 21 after MI should be the best timing to inject MSCs for achieving the highest MSC engraftment efficiency.

## Discussion

We demonstrated the immunomodulation of MSC mechanosensing to regulate MSC engraftment in the heart infarct zone after MI, providing evidences of mechano-immune coupling-dependent cell adhesion on different stiffness substrates.

Immobilized IL-6 strengthens MSC adhesion on rigid substrate through integrin α_5_β_1_ signaling axis rather than α_v_β_3_’s. Integrin α_5_β_1_ activates myosin-II-dependent force generation and substrate mechanosensing to induce nascent adhesion assembly (Galbraith et al., 2007; Schiller et al., 2013), while integrin α_v_β_3_ enables mechanotransduction that results in remodeling of FAs and the directional orientation of the actin cytoskeleton (Roca-Cusachs et al., 2009; Schiller et al., 2013). The selective activation of integrin α_5_β_1_ indicated by immobilized IL-6 suggests its essential role in regulating the early stage of MSC mechanosensing, specifically in regulating MSC adhesion on a rigid substrate by promoting nascent adhesion formation. Thus, our data further imply that the decline of inflammatory cytokine level in the later phase after MI may impair MSC mechanosensing and engraftment on the rigid infarct zone.

We demonstrated that the immobilized IL-6-induced enhancement of MSC adhesion was dependent on phosphatase SHP2. SHP2 has been reported to regulate both integrin α_5_β_1_’s affinity and avidity to FN. On one hand, SHP2 dephosphorylates and activates Rho-associated protein kinase II (ROCKII) (Lee et al., 2013), the main effector enabling myosin-II-mediated force generation (Sun et al., 2019), to further inside out strengthen the force-dependent binding between integrin α_5_β_1_ and FN through catch bond (Kong et al., 2009, 2013). On the other hand, SHP2 promotes integrin β_1_ clustering (avidity) in a lipid raft-dependent manner and accelerates cell spreading (Lacalle et al., 2002). Targeting SHP-2 to lipid raft domains induces integrin β_1_ clustering (Lacalle et al., 2002), which enhances bond lifetimes with FN and clustering sizes with increasing substrate stiffness to further promote focal adhesion kinase (FAK) Y397 phosphorylation and mechanosensing (Cheng et al., 2020). Furthermore, SHP2 has been implicated in signaling pathways initiated by inflammatory mediators other than IL-6, suggesting its more general role of immune-mechano coupling in regulating cell adhesion. Together, these results demonstrate that SHP2 can be activated by immobilized IL-6 and then transduce inside-out signaling to regulate the affinity and avidity of integrin α_5_β_1_ for enhancing MSC mechanosensing, adhesion, and engraftment in the infarct zone.

Inflammatory cytokines are released in a soluble form and can also be in immobilized form by binding to the N-terminal of FN in the infarct region (Alon et al., 1994; Frangogiannis, 2014). Our result showed that only immobilized IL-6 promoted MSC adhesion under shear flow. We speculate that immobilized IL-6 enhances MSC adhesion from three aspects. Firstly, immobilized IL-6 may provide additional anchorage site other than FN/integrin linkages for MSC adhesion, helping stabilize MSC and substrate adhesion and facilitate mechanical force to induce integrin’s conformational change and outside-in activation of integrin (Chen et al., 2012). Secondly, the immobilized form may enable force transduction from IL-6 receptor to its downstream signaling molecules (e.g., SHP2), activating potential mechano-regulation-dependent IL-6/SHP2 signaling pathway. Supporting this speculation, SHP2 has been reported to be mainly recruited and activated in membrane ruffles (Sun et al., 2013), the peripheral cellular constructions bearing large force and under which nascent adhesions assemble (Giannone et al., 2007). The force-bearing membrane ruffles localization and activation implies potential force regulation of SHP2 activity. Thirdly, immobilized IL-6/SHP2 signaling may be triggered in immediate proximity to FN occupancy that can temporally and physically couple inside-out and outside-in bidirectional activation of integrin α_5_β_1_ to accelerate MSC adhesion on rigid substrate. In contrast, a global signal induced by soluble IL-6 fails to induce the full bidirectional activation of integrin.

In addition to IL-6, we also observed the effect of IL-1β and TNF-α on enhancing MSC adhesion. While both IL-1β and TNF-α have been reported to promote cell adhesion through upregulating expressions of FA proteins, we did not observe their effect on MSC adhesion under flow (Xia et al., 1998; Rajshankar et al., 2012). One possible explanation for this discrepancy is that our investigation of MSC adhesion under shear flow is restricted to a short contact duration mainly involving interaction between integrin and FN rather than FA remodeling and connection to the actin cytoskeleton.

*In vivo*, the temporal coincidence of enhancement of MSC engraftment with inflammatory cytokine upregulation and tissue stiffness increase indicates that simultaneous modulation of the inflammatory and mechanical environment may further promote MSC engraftment into the infarct heart after MI. Despite a relatively favorable inflammatory environment early after MI, the mechanical property of the infarcted myocardium is unsuited for MSC adhesion. Mimicking infarcted heart stiffness *in vitro*, we showed that MSC adhesion strengthens with increasing substrate stiffness, implying that the relatively softer tissue stiffness at the earlier phase after MI hinders MSC adhesion and engraftment. Encapsulating MSCs in hydrogel with tunable mechanical properties has been shown to improve MSC engraftment into the heart (Burdick et al., 2016), whose effect may be partially attributed to overcoming the mechanical limitation of soft tissue stiffness on MSC adhesion at the early stage. However, hydrogel may isolate MSCs from its surrounding inflammatory environment and block the biochemical signal stimulation. This defect could be overcome by modification of hydrogel with IL-6 coating to promote MSC adhesion. In addition, small molecule intervention that targets signaling molecules such as SHP2 may have the same effect.

What is more interesting, our *in vivo* immunohistochemical staining results showed a very similar alteration of the number of infiltrated macrophages on different days after MI as the upregulation of inflammatory cytokines, suggesting potential regulation of macrophage adhesion to the infarcted heart tissue by inflammatory cytokines. In fact, our *in vitro* flow chamber assay showed that immobilized IL-6 promoted the adhesion of the macrophage cell line RAW264.7 cells to FN-coated substrate, and this adhesion enhancement effect was blocked by both α_5_β_1_ blocking mAb and shp099 (Supplementary Figure 7), demonstrating a more general role of immobilized IL-6/SHP2/α_5_β_1_ signaling axis on cell adhesion in addition to MSCs. As inflammatory cytokines are mainly produced by immune cells including macrophages, this immobilized IL-6-mediated adhesion enhancement may provide a positive feedback mechanism to promote immune cell infiltration into the infarct region and augmentation of immune response after MI. Combined with our result that only immobilized IL-6 but not its soluble counterpart promotes cell adhesion on stiffened substrate, these data further indicate a potential mechano-regulation of immune response mechanism that have been reported on other immune receptors (Chen and Zhu, 2013; Wu et al., 2019; Zhu et al., 2019).

Taken together, we revealed inflammatory cytokine couples with substrate stiffness can cooperatively modulate MSC mechanosensing, adhesion *in vitro*, and engraftment *in vivo* after MI. If MSC transplantation strategies can cooperatively modulate the mechanical and inflammatory environment for better MSC adhesion, the efficiency of MSC engraftment to benefit clinical MI treatment might be improved.

## Data Availability Statement

All datasets presented in this study are included in the article/Supplementary Material.

## Ethics Statement

The animal study was reviewed and approved by the Institutional Animal Care and Use Committee of Zhejiang University.

## Author Contributions

WC, JW, and XH conceived and designed the project. DZ performed AFM, MI induction, and MSC transplantation experiments. PW performed PCR and immunohistochemical experiments. CX performed flow chamber assay. DZ, PW, WH, and TZ performed data analysis. WC, DZ, and PW wrote the manuscript. All authors contributing to its revision.

## Conflict of Interest

The authors declare that the research was conducted in the absence of any commercial or financial relationships that could be construed as a potential conflict of interest.
