# Supplementary material for: Inflammatory Cytokines Alter Mesenchymal Stem Cell Mechanosensing and Adhesion on Stiffened Infarct Heart Tissue After Myocardial Infarction
Source: Front Cell Dev Biol. 2020 Oct 23;8:583700. doi: 10.3389/fcell.2020.583700 (PMC7645114; doi:10.3389/fcell.2020.583700)
Supplement: Supplementary file 1 [file Data_Sheet_1.pdf]

Supplementary Material

1 Supplementary Table

	Polyacrylamide gel stiffness (E)			
	6.7kPa	25.1kPa	45.7kPa	2.3kPa
**Stock solution**				
40% Acrylamide (mL)	3.75	2.34	3.00	3.00
2% Bisacrylamide (mL)	0.75	1.88	0.75	1.40
dH_2O (mL)	0.5	0.78	1.25	0.60
Total volume(mL)	5	5	5	5
**Working solution**				
Stock solution (µL)	125	200	250	250
10% Ammonium persulfate (µL)	2.5	2.5	2.5	2.5
TEMED (µL)	2.5	2.5	2.5	2.5
ddH_2O (µL)	372	297	247	247

2 Supplementary Figures

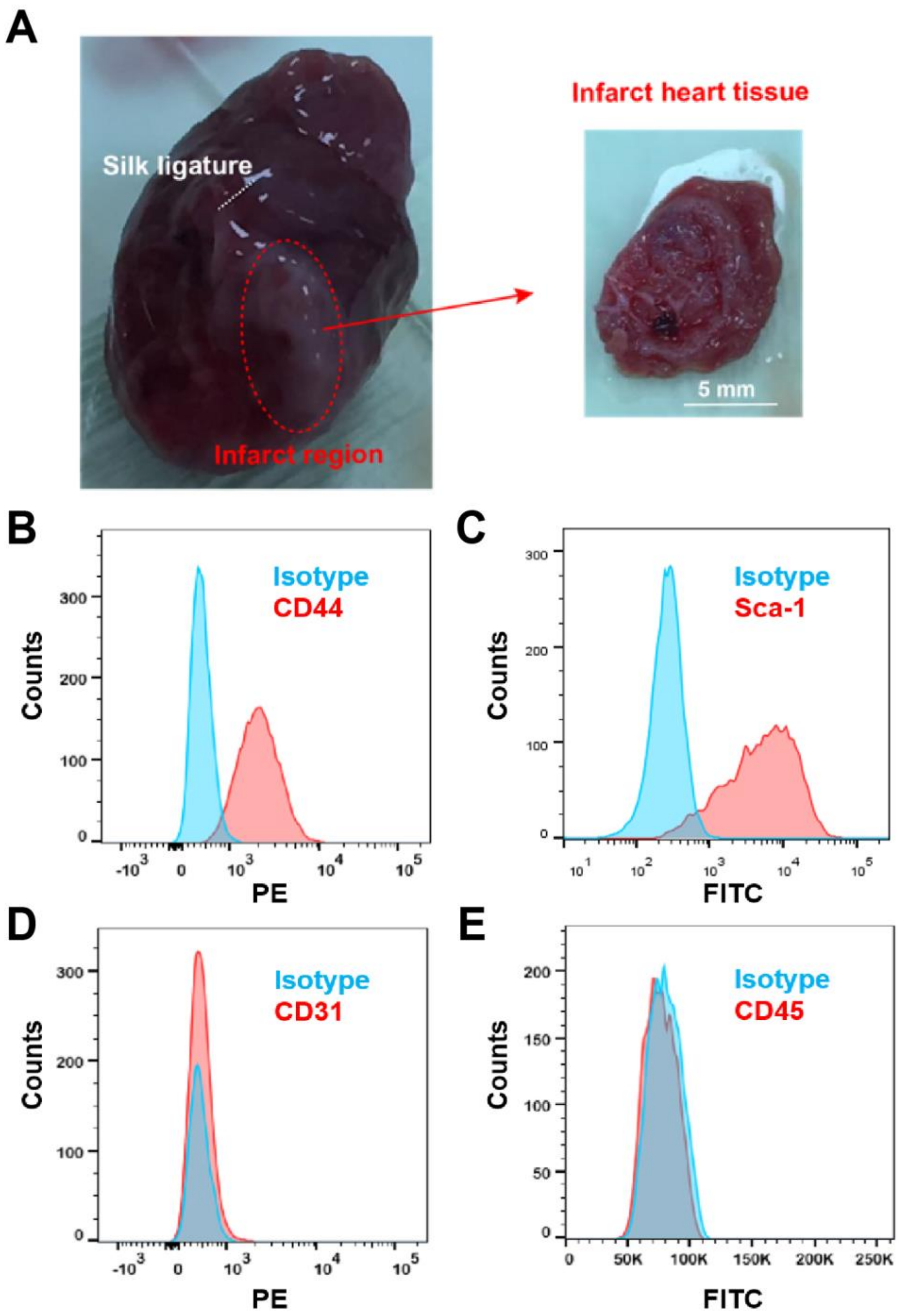

Supplementary Figure 1. Tissue block from infarcted heart for AFM probing and characterization of MSC surface markers by flow cytometry. (A) Tissue from the infarct region was cut into small blocks for AFM probing. The silk ligature is indicated by white dotted line and the infarct region from which tissue blocks were obtained indicated by red dotted circle. **(B-D)** MSCs were characterized for their surface antigens expression of CD44, Stem-cell antigen 1 (SCA-1), CD31 and CD45 with flow cytometry.

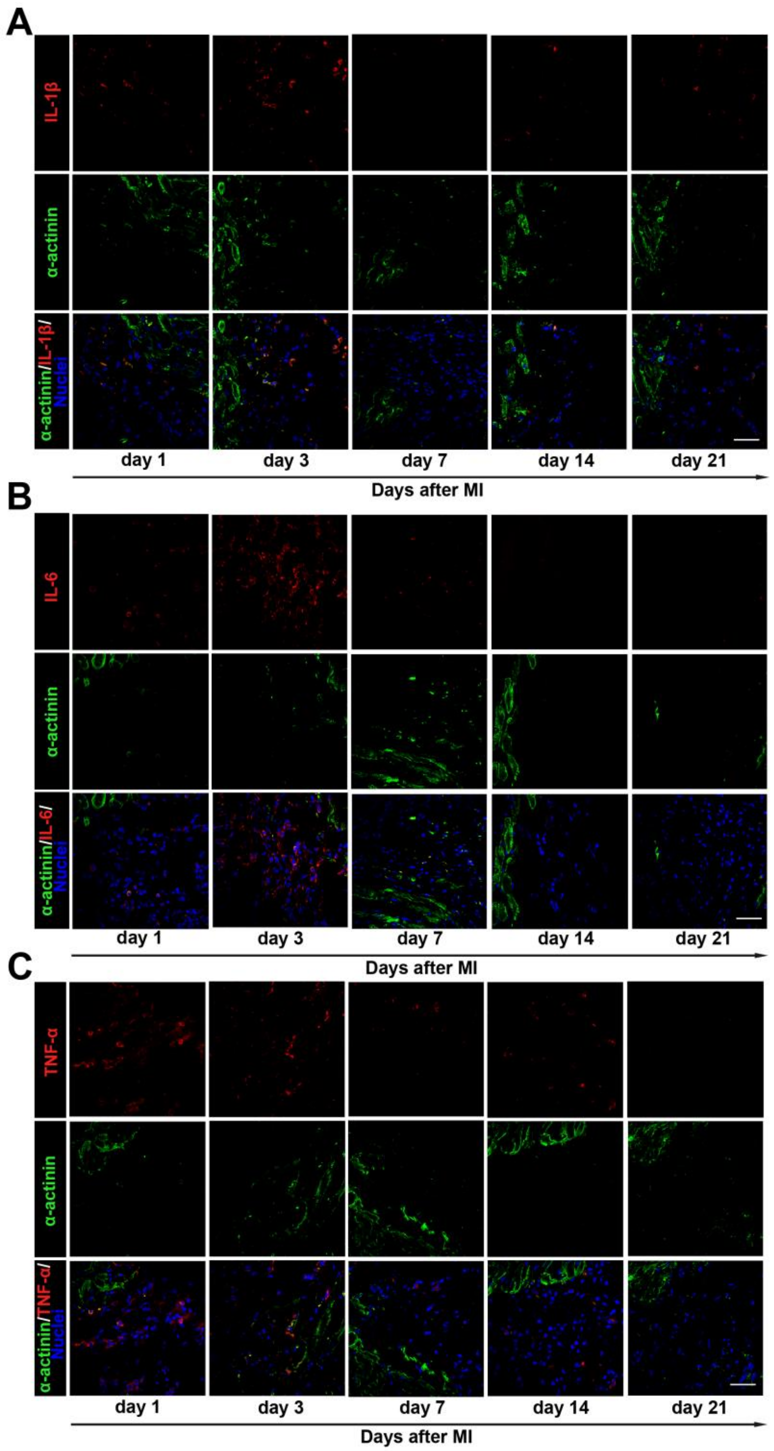

Supplementary Figure 2. Upregulation of inflammatory cytokines in the peri-infarct region of the rat heart early after MI. Representative images of immunohistochemical co-staining of IL-1β **(A)**, IL-6 **(B)**, and TNF-α **(C)** with α-actinin in the peri-infarct region of the rat heart at different times after MI. Nuclei were stained with Hoechst 33258. Scale bars refer to 40 μm.

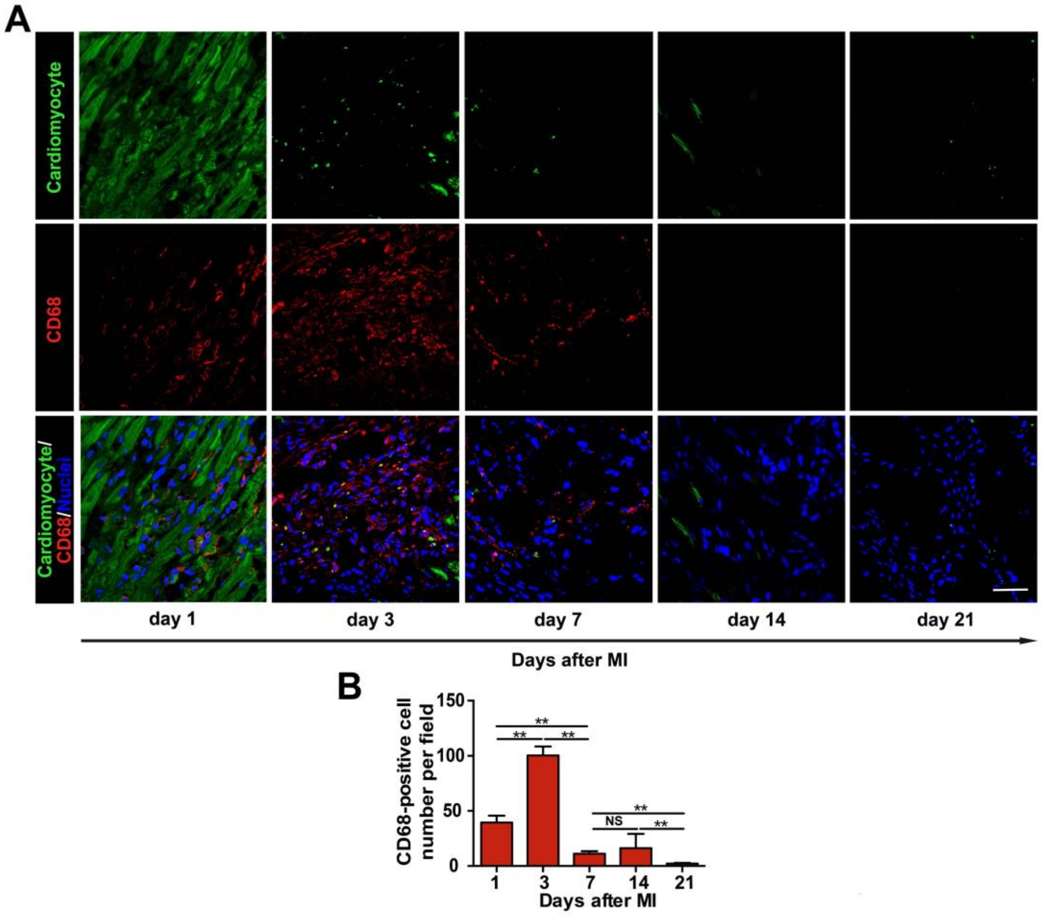

Supplementary Figure 3. Macrophage infiltration into the infarct region of the rat heart at the early acute phase after MI. (**A)** Representative immunofluorescence images of macrophage infiltration in the infarct region at different times after MI **(A)**. CD68 was used to mark macrophage. Nuclei were stained with Hoechst 33258. (**B)** Comparisons of average numbers of $CD68^+$ cells per field. Scale bars in **(A)** refer to 40 μm. ** and NS in **(B)** refer to $p < 0.01$ and no significance respectively. N=3 rats for each time point. Error bars in **(D)** represent SEM of three repeats.

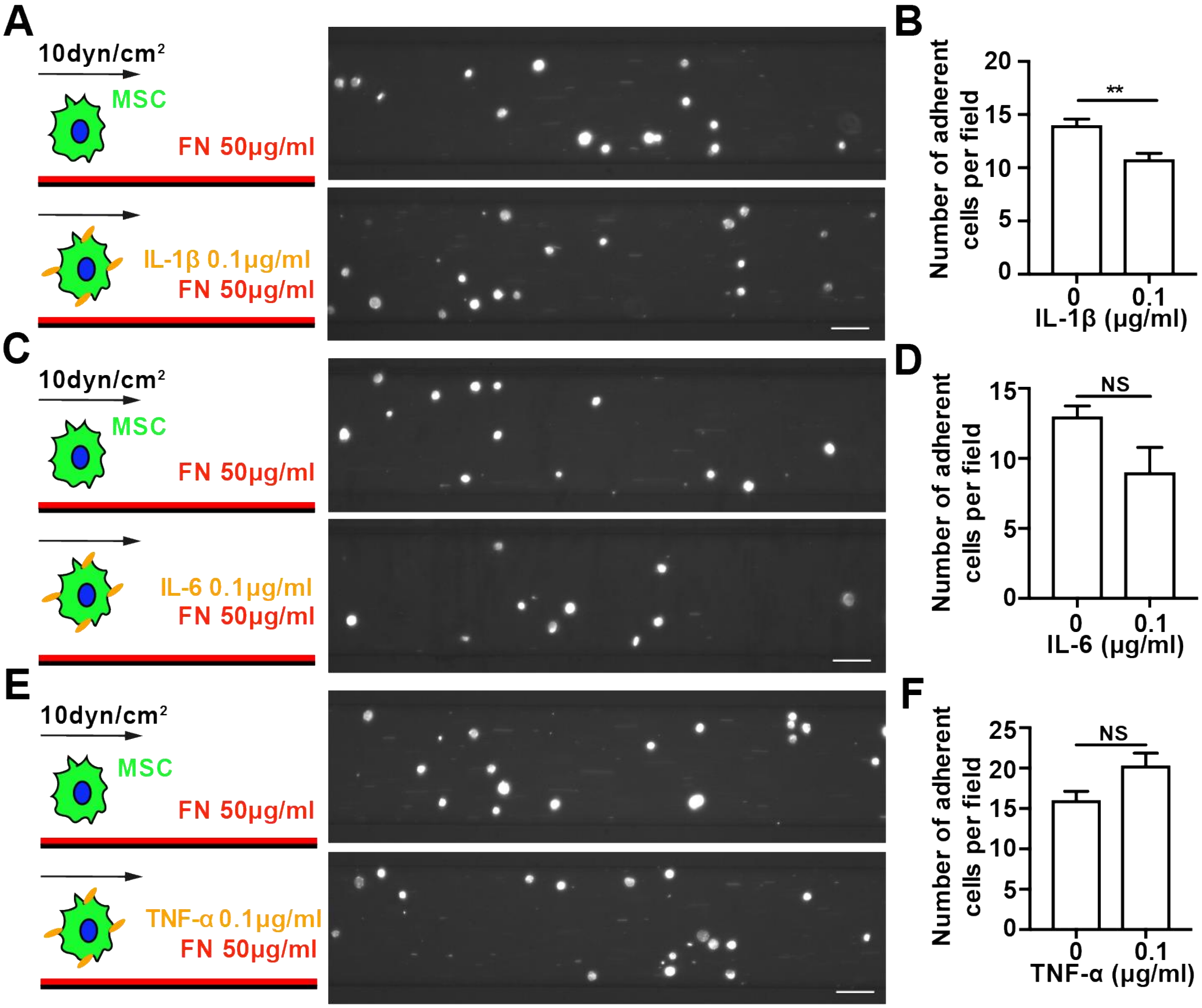

Supplementary Figure 4. Effects of soluble inflammatory cytokines on MSC adhesion under shear flow. Representative images of attached MSCs pretreated with 0.1µg/mL of IL-1 β **(A)**, IL-6 **(C)** or TNF-α **(E)** for 30min before perfusion over microfluidic channels coated with 50 µg/mL of FN, and respective average numbers of adherent MSCs per field are shown in **(B)**, **(D)** and **(F)**. Scale bars in **(A)**, **(C)** and **(E)** refer to 200 µm. ** and NS in **(B)**, **(D)** and **(F)** refer to $p < 0.01$ and no significance respectively. Error bars in **(B)**, **(D)** and **(F)** represent SEM of three, four and six repeats respectively.

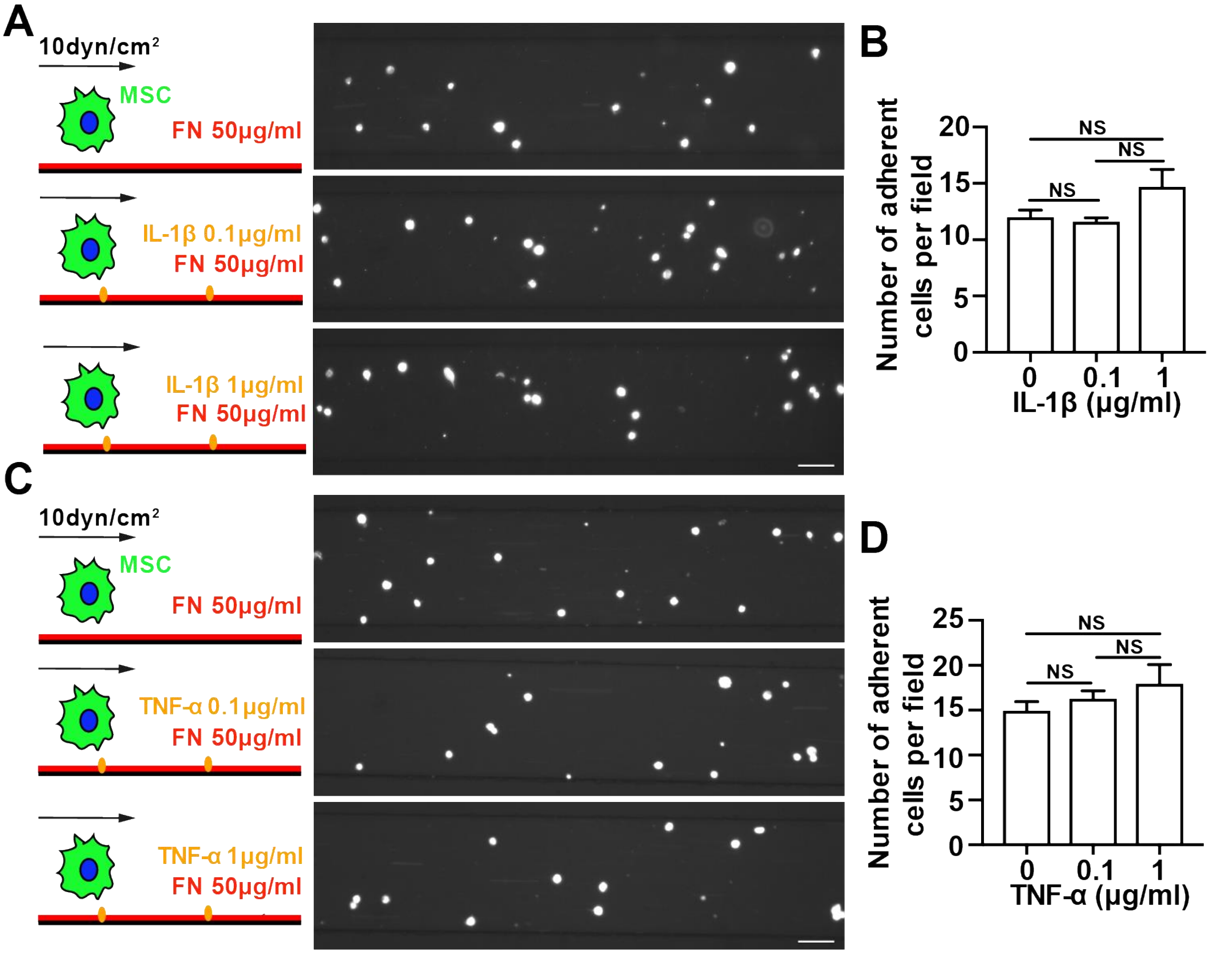

Supplementary Figure 5. Immobilized IL-1β and TNF-α have no effect on enhancing MSC adhesion under shear flow. Representative images of MSCs perfused over microfluidic channels coated with 50 µg/mL of FN combined with increasing concentration of IL-1β **(A)** or TNF-α **(C)**, and respective average numbers of adherent MSCs per field are in **(B)** and **(D)**. Scale bars in **(A)** and **(C)** refer to 200 µm. NS in **(B)** and **(D)** refers to no significance. Error bars in **(B)** and **(D)** represent SEM of three repeats.

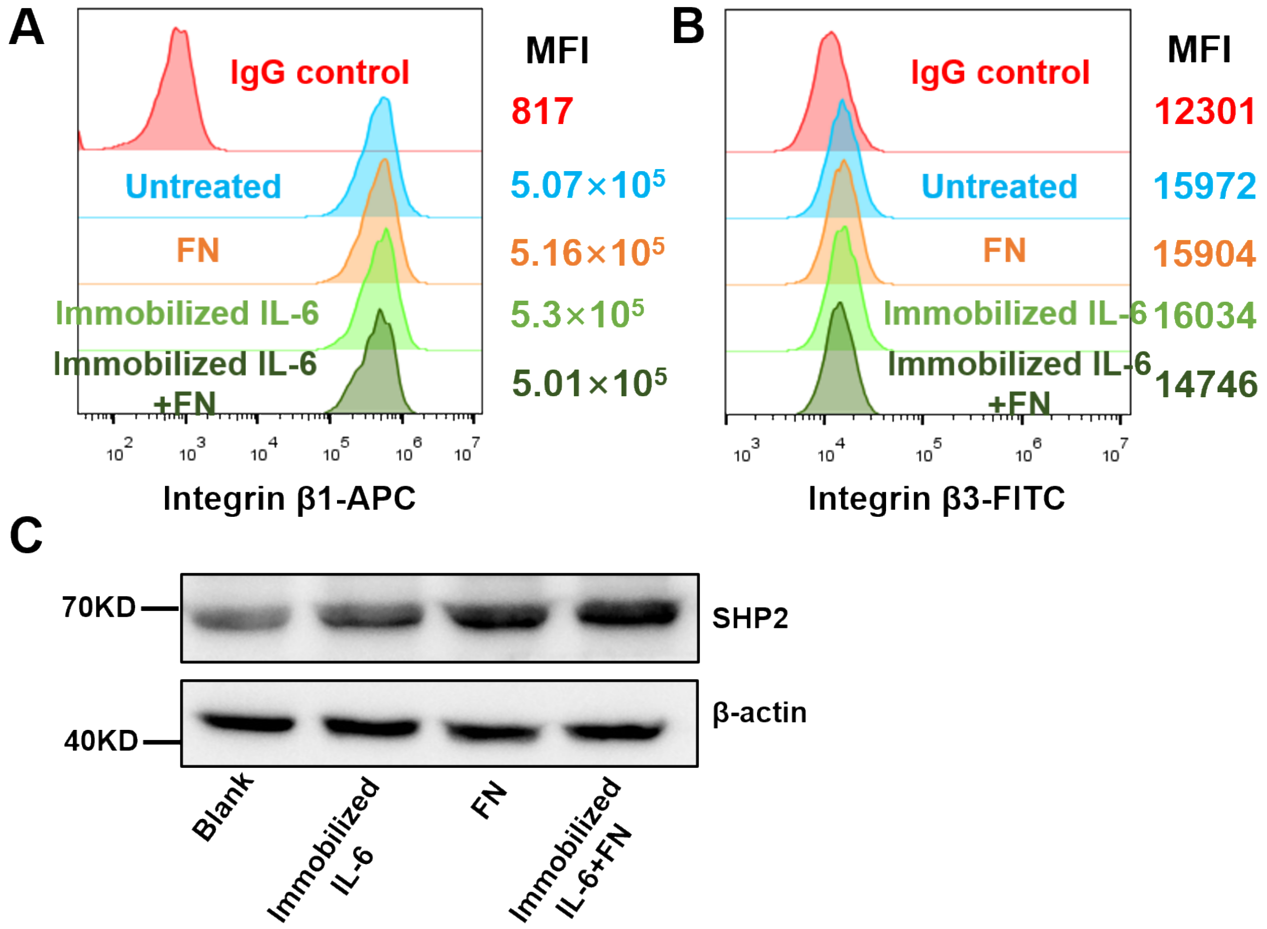

Supplementary Figure 6. The effect of immobilized IL-6 on integrin and SHP2 expression. MSCs were stimulated with FN, immobilized IL-6 or FN combined with immobilized IL-6 respectively. The expression level of integrin β_1 (**A**) and integrin β_3 (**B**) on MSC surface under different conditions were detected by flow cytometry. The values of mean fluorescence intensity (MFI) for each group were shown on the right. The expression level of SHP2 under different conditions were detected by western blot (**C**). β-actin levels were also evaluated to serve as a control.

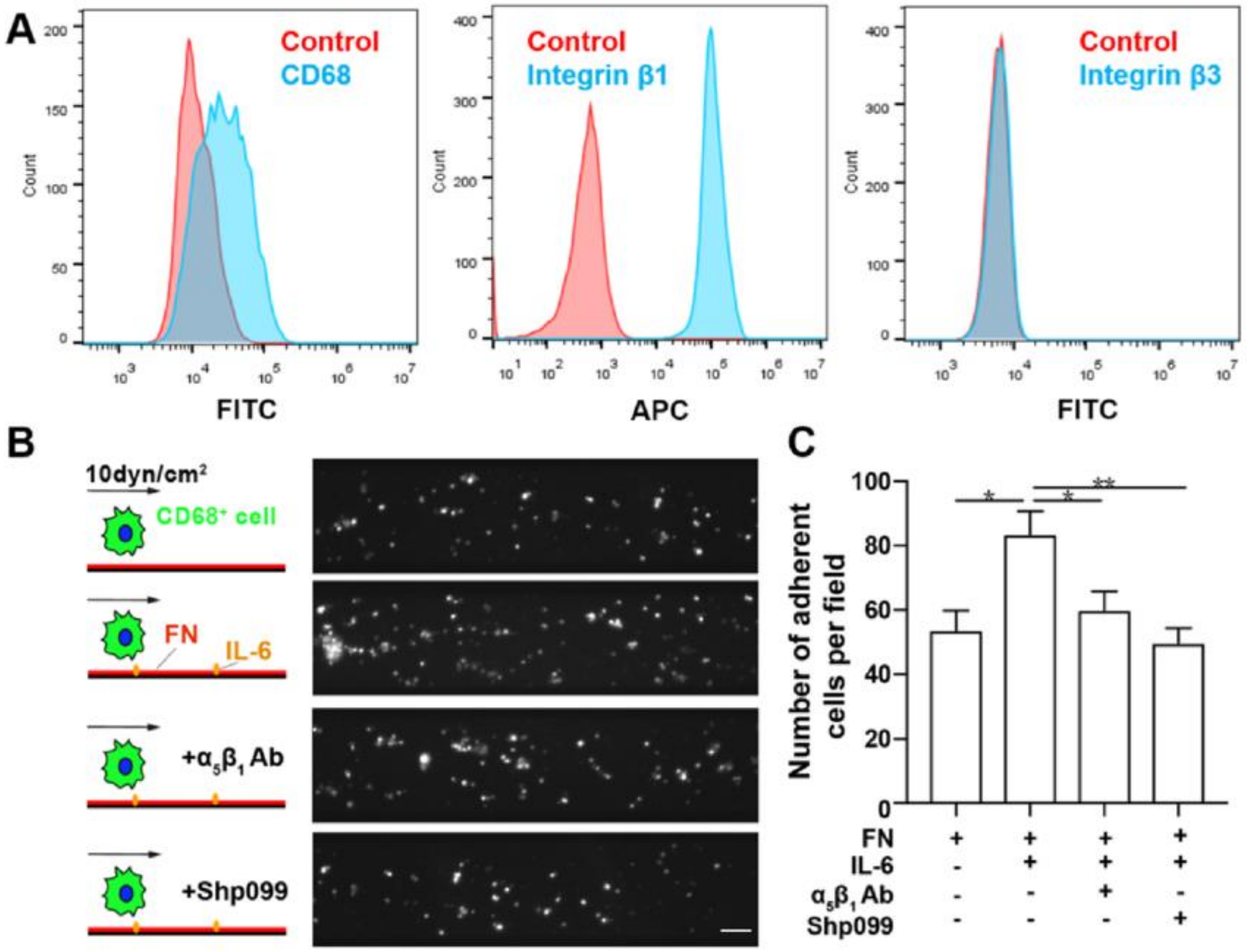

Supplementary Figure 7. The effect of immobilized IL-6/SHP2/$\alpha_5\beta_1$ signaling axis on macrophage cell adhesion. (A) The expression of CD68, integrin β_1 and β_3 on the surface of macrophage cell line RAW264.7 was determined by flow cytometry. **(B)** Representative images of attached RAW264.7 cells pretreated with $\alpha_5\beta_1$ blocking mAb or shp099 for 30min before perfusion across FN coated microfluidic channels in the presence of immobilized IL-6. The corresponding average numbers of adherent RAW264.7 cells per field were calculated and shown in **(C)**. Scale bars in **(B)** refer to 200 μm. ** and * in **(C)** refer to $p < 0.01$ and $p < 0.05$ respectively. Error bars in **(C)** represent SEM of three repeats.
